# Supplementary material for: Exosomal Small RNA Sequencing Profiles in Plasma from Subjects with Latent Mycobacterium tuberculosis Infection
Source: Microorganisms. 2024 Jul 12;12(7):1417. doi: 10.3390/microorganisms12071417 (PMC11278582; doi:10.3390/microorganisms12071417)
Supplement: Supplementary file 1 [file microorganisms-12-01417-s001.zip › TABLE S1.pdf]

Table S1 The basic statistics for small RNA-seq reads generated from plasma-derived exosomes of tuberculosis patients and healthy control.

Sample	Sequence Type	Raw Tag Count	Low Quality Tag Count	Short Valid Length Tag	Clean Tag Count	Q20 of Clean Tag	Percentage of Clean Tag (%)
HC-1	SE50	37748736	170621	2183440	34463894	98.5	91.3
HC-2	SE50	37748736	166071	50355	37275940	98.4	98.75
HC-3	SE50	37748736	164426	671437	36282512	98.7	96.12
HC-4	SE50	37748736	177984	1630014	35208210	98.6	93.27
LTBI-1	SE50	37748736	164910	849322	36196486	98.6	95.89
LTBI-2	SE50	37748736	165927	37080965	37080965	98.7	98.23
LTBI-3	SE50	37748736	162207	787206	36369562	98.7	96.35
LTBI-4	SE50	37748736	164729	536931	36541720	98.7	96.8
